# Maneuvering investigation of theoretical and experimental parameters for Al-doped Cu (In, Ga) Se_2_ thin film solar cells with and without a back surface field layer

**DOI:** 10.1039/d5ra03970c

**Published:** 2025-09-05

**Authors:** A. Ashok, Karthick Sekar, D. Acosta, Hind Saeed Alzahrani, Amani H. Alfaifi, Talaat A. Hameed

**Affiliations:** a Sección de Electrónica de Estado Sólido (SEES), Departamento de Ingeniería Eléctrica Cinvestav, San Pedro Zacatenco C.P. 07360 Mexico City Mexico ashokad@cinvestav.mx; b Aix-Marseille Université, CNRS, IM2NP, Faculté de Saint Jérôme 13397 Marseille Cedex 20 France; c Department of Condensed Matter, Institute of Physics, Universidad Nacional Autónoma de México (UNAM) Couoacán C.P. 04510 Mexico City Mexico; d Department of Physics, College of Science, Taif University P.O. Box 11099 Taif 21944 Saudi Arabia; e Solid-State Physics Department, Physics Research Institute, National Research Centre 33 El Bohouth St., Dokki Giza 12622 Egypt Talaathameed83@gmail.com Talaathamid@yahoo.com

## Abstract

Aluminum-doped copper indium gallium selenide/sulfide (CIGAS) is a favorable absorber material for solar cell applications; however, the number of reports on CIGAS solar cells currently remains limited. In this study, we therefore employed SCAPS-1D software for the theoretical modeling of CIGAS thin film solar cells and investigated the effect of material properties and device configurations on solar cell photovoltaic (PV) parameters. Initially, key parameters such as thickness and charge carrier concentrations of each layer used in CIGAS PV devices were studied and optimized to obtain suitable conditions for high device performance. The impact of the various buffer window layers (BWL)—such as CdS, In_2_S_3_, ZnS, ZnSe, In_2_Se_3_, V_2_O_5_, ZnO, and MgZnO—as well as the back surface field (BSF) layers, including Sb_2_Se_3_, AlSb, CuGaSe_2_, SnS, BaSi_2_, MoS_2_, MoSe_2_, p-Si, CuS, and WSe_2_, was systematically tested to determine a CIGAS solar cell configuration with greater efficiency. After meticulous optimization, CdS and Sb_2_Se_3_ materials were selected as the best BWL and BSF layers for the CIGAS device configuration, respectively, demonstrating a maximum power conversion efficiency (PCE) of 32.2% compared to other chosen materials. Finally, an experimentally obtained CIGAS absorber and CdS buffer material properties were introduced into optimized conditions with and without a BSF layer to further analyze their influence on solar cell performance. This also confirmed that the BSF layer significantly boosts device efficiency compared to the conventional CIGAS device.

## Abstract 2

Aluminum-doped copper indium gallium selenide/sulfide (CIGAS) is a favorable absorber material for solar cell applications; however, the number of reports on CIGAS solar cells currently remains limited.

## Graphical abstract

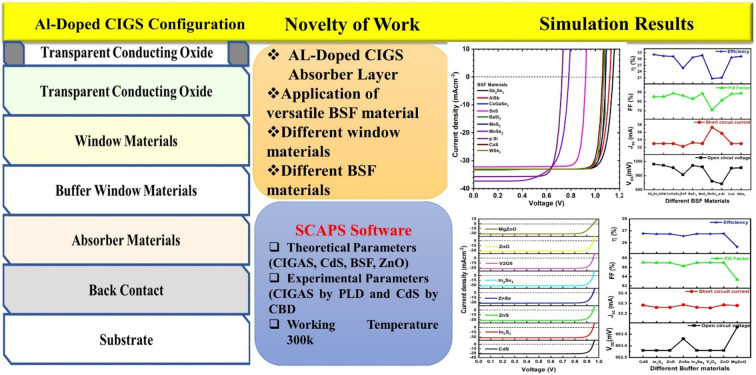

## 1. Introduction

Over the past several decades, extensive research has focused on developing cost-effective and high-efficiency photovoltaic (PV) devices for harvesting solar energy. Based on semiconducting materials, PV technology is classified into four main generations: first (silicon and III–V), second (CIGS, a-Si, and CdTe), third (perovskite, dye-sensitized, quantum-dot, and multi-junction), and fourth (carbon-based materials, nanomaterials, and hybrid).^1–4^ In second-generation solar cells (also known as thin film solar cells), the efficiency-to-cost ratio of PV devices has improved by using novel and inexpensive growth techniques and materials.

Polycrystalline copper indium gallium aluminum selenide/sulfide (CIGAS) thin film solar cells have the potential to achieve high efficiency at a lower device cost and are suitable for portable, terrestrial, and space applications.^5,6^ These solar cells are potentially attractive in PV technology because of various characteristics, such as direct and flexible bandgap, high absorption coefficient, ease of manufacturing, flexibility and versatility in device design, and high stability. To date, many research groups have manufactured CIGAS-based thin film solar cells with efficiency greater than 20% on a laboratory scale.^7–10^

CIGAS-based solar cells reached the highest efficiency of 23.6% for a single solar cell in an area of approximately 1 cm^2^ by the Evolar/UppsalaU group and 20.3% efficiency for 100 cells in an area of 527 cm^2^ by Avancis.^11,12^ This record efficiency for CIGAS-based thin film solar cells is comparatively less than that of first-generation solar cells. However, the performance of CIGAS-based PV devices can be further enhanced by understanding the material properties and device structures, exploring novel and inexpensive materials, analyzing the effect of defects (*i.e.*, grain boundaries and vacancies), doping the CIGAS material with different elements, and other strategies.^13,14^ A significant challenge for the commercialization of CIGS-based PV devices is their reliance on the use of rare and more expensive elements (*i.e.*, indium and gallium).

Modifying the properties of materials and structures of CIGAS PV devices can resolve earlier problems, for example, by introducing aluminum in the CIGAS absorber material. Aluminum, a group III element, exhibits properties similar to those of indium and gallium. Aluminum is lightweight, easy to manipulate, cost-effective, non-toxic, fully recyclable without loss of properties, and highly conductive, making it a versatile and promising choice for CIGAS absorber materials.^15^ In addition, using Al in CIGS significantly impacts the bandgap of the CIGAS absorber material, carrier transport properties, density of defects, and recombination centers at the interface and in bulk materials.^15^ The bandgap of the CIGAS material can be tuned from 1.04 eV (for CuInSe_2_) to 2.7 eV (for CuAlSe_2_), relying on the gallium and aluminum content in the material.^16,17^ Because efficient CIGS PV devices have been obtained within a specific absorber bandgap range (1.1–1.5 eV), doping CIGS with Al enables better utilization of the solar spectrum by optimizing light absorption for the corresponding CIGAS bandgap. The application of Al in III–V solar cells has significantly improved two main parameters—the open-circuit voltage (*V*_oc_) and short circuit current density (*J*_sc_)—and consecutively enhanced the power conversion efficiency (PCE) of solar cells.^18,19^

Our group extensively reviewed the effect of Al on the structure, as well as optical and mechanical properties of CIGS.^20,21^ We found that the optical band gap of CIGS prepared by a one-step sputtering process reached 1.68 eV with Al doping at 7 atomic percent (at%) for 200-nm-thick films. In our related research, we were interested in the impact of thickness, bandgap, and carrier concentration of CIGS, and determined that the theoretically optimum bandgap for CIGS absorber material was 1.4 eV.^22^ Although Al can be readily incorporated into the CIGS matrix using various facile methods and techniques, and while its material properties have been investigated,^23–25^ there is a notable lack of information regarding the solar cell configuration for Al-doped CIGS, including buffer layers, back surface field layer, and upper and lower electrodes. Therefore, performing theoretical modeling before beginning experimental activities can be helpful in optimizing the properties of materials and designing efficient novel devices.

Theoretical modeling plays a vital role in interpreting the physical behaviors and circumstances of semiconductor devices for rapid improvement in design and efficiency. Many simulators are available for modeling various PV devices in three different dimensions (*i.e.*, 1D, 2D, and 3D).^26–28^ The one-dimensional solar cell capacitance simulator (SCAPS-1D) is freely available windows-oriented software that can provide useful information related to the properties of materials and PV devices. SCAPS-1D software was originally developed to analyze CIGAS-based thin film solar cells.^29,30^

Recently, the study area of SCAPS-1D has increased to simulate different types of PV devices, such as c-Si, the III–V family, a-Si, CdTe, CZTS, perovskite, organic, tandem, multi-junction, and others.^31–41^ Because of these advancements in the features of SCAPS-1D software, it is proficient in modeling and optimizing PV devices. Various properties, such as thickness, bandgap, electron affinity, dielectric constant, carrier density, carrier mobility, absorption coefficient, and defect density of materials used in solar cells, can be thoroughly examined using SCAPS-1D software.

In the current study, newly proposed materials and structures (CIGAS with and without back surface field (BSF) materials) for CIGS-based solar cells were proposed and investigated using SCAPS-1D software. The properties of the CIGAS absorber, CdS buffer, ZnO window, and BSF layers were graded to obtain maximum efficiency for the CIGAS thin film solar cells. The numerical analysis not only focused on the optimization process to achieve the maximum efficiency possible but also on the impact of the BSF layer on device performance. In addition, theoretical modeling was performed for different types of buffer materials and BSF materials to determine the most optimal alternative materials for CIGAS PV devices. Furthermore, to compare the results between theoretical and experimental parameters, the experimentally obtained parameters of the CIGAS absorber and the CdS buffer layers were introduced to the theoretically optimized conditions. This simulation study supports the comparison of results for theoretical and experimental parameters and then determining their optimal conditions for high-efficiency PV devices.

## 2. Simulation details

The SCAPS-1D software was initially developed by researchers from the University of Ghent—Marc Burgelman, Koen Decock, and Alex Niemegeers—for the theoretical modeling of CIGAS-based PV devices. A Gummel iteration scheme with Newton–Raphson sub-steps is normally used in this software.^42^ This assists in solving the Poisson and continuity equations for charge carriers, which are crucial for understanding the electric field distribution of solar cells. This software is a windows-oriented program that allows the theoretical design of efficient PV devices by exploring material properties, performance characteristics, and device architectures.

**Fig. 1:**
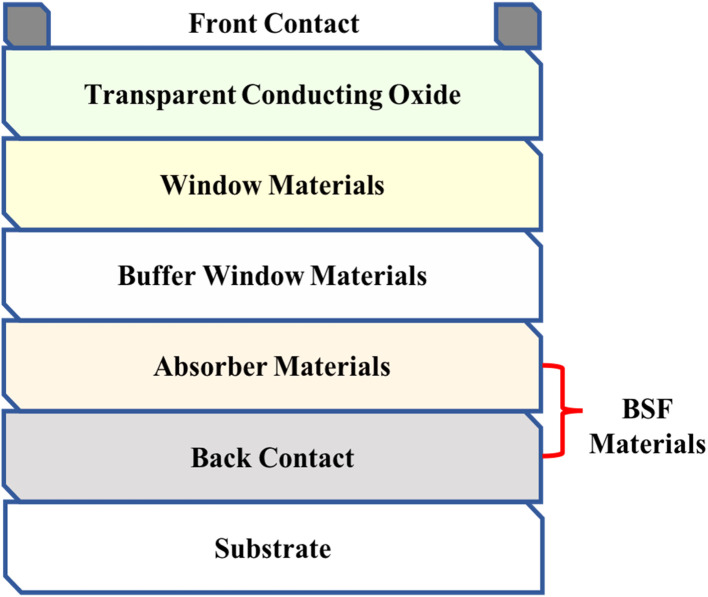
Schematic of the CIGAS solar cell structures used in the SCAPS-1D simulator.

Apart from front and back contacts, SCAPS-1D software is designed for up to 7 layers of solar cells.^43^ This software can also provide various characteristics, including solar cell parameters, quantum efficiency (QE), recombination profiles, band diagrams, tunneling mechanism, and admittance measurements.^44^ In the current study, SCAPS-1D software was utilized to analyze the effect of material properties and solar cell structures on device performance. In addition, the theoretical and experimental parameters of materials used in CIGAS thin film solar cells with and without a BSF layer were also explored. Four solar cell parameters, namely *V*_oc_, *J*_sc_, fill factor (FF), and PCE, were estimated to understand the device performance under different conditions. Fig. 1 displays the structure of CIGAS thin film solar cells introduced in SCAPS-1D, where the BSF layer is employed between the CIGAS absorber layer and the back contact.

During the simulation process, standard conditions with a working temperature of 300 K and a solar spectrum of AM 1.5 G were used. The theoretical parameters of the CIGAS absorber layer, CdS buffer layer, ZnO window layer, and BSF layer were obtained from various studies^29,30,45–47^ and are presented in Table 1. The thickness and carrier concentration (bandgap only for CIGAS) for these materials were graded to obtain the maximum efficiency possible. The influence of different types of buffers and BSF materials on the solar cell parameters was also thoroughly investigated (see Table 2).^48–56^ Finally, the experimental parameters for CIGAS absorber materials (obtained by pulsed laser deposition and magnetron sputtering) and CdS buffer materials (synthesized by chemical bath deposition) were addressed in theoretically optimized CIGAS PV devices with and without a BSF layer. All these variations in solar cells were generated to identify cost-effective designs and materials with improved device performance.

**Table 1:** Parameters of each material used in CIGAS solar cells^29,30,45–47,57^

Material parameters	CIGAS absorber	BSF material	CdS buffer	ZnO window	ZnO : Al TCO
Thickness (nm)	200–6000	20–400	20–300	20–500	50
Bandgap (eV)	1.00–1.50	1.4	2.45	3.25	3.5
Electron affinity (eV)	4.5–4.0	4.1	4.2	4.55	4.5
Dielectric permittivity	13.6	13.6	10	9	9
CB density of states (cm^−3^)	2.2 × 10^18^	2.2 × 10^18^	2.2 × 10^18^	2.2 × 10^18^	2.2 × 10^18^
VB density of states (cm^−3^)	1.8 × 10^19^	1.8 × 10^19^	1.8 × 10^19^	1.8 × 10^19^	1.8 × 10^19^
Electron thermal velocity (cm s^−1^)	1 × 10^7^	1 × 10^7^	1 × 10^7^	1 × 10^7^	1 × 10^7^
Hole thermal velocity (cm s^−1^)	1 × 10^7^	1 × 10^7^	1 × 10^7^	1 × 10^7^	1 × 10^7^
Electron mobility (cm^2^ V^−1^ s^−1^)	100	100	100	100	100
Hole mobility (cm^2^ V^−1^ s^−1^)	25	25	25	25	25
Donor density (cm^−3^)	0	0	10^15^–10^19^	10^15^–10^19^	10^17^
Acceptor density (cm^−3^)	10^12^–10^20^	10^12^–10^20^	0	0	0

**Table 2:** Parameters of different buffer materials and BSF materials^48–56^

Buffer Material Parameters	Bandgap (eV)	EA (***χ***) (eV)	DC (***ε***)	Parameters BSF Materials	Bandgap (eV)	EA (***χ***) (eV)	DC (***ε***)
CdS	2.45	4.20	10.00	Sb_2_Se_3_	1.62	4.00	7.08
In_2_S_3_	2.82	4.50	13.50	AlSb	1.60	3.60	10.90
ZnS	3.68	3.50	10.00	CuGaSe_2_	1.70	3.80	13.60
ZnSe	2.9	4.10	10.00	SnS	1.25	4.20	12.50
In_2_Se_3_	2.40	4.00	13.6	BaSi_2_	1.30	3.30	11.17
V_2_O_5_	2.40	4.50	10.00	MoS_2_	1.62	4.20	13.60
ZnO	3.25	4.55	9.00	MoSe_2_	1.10	3.80	14.30
MgZnO	3.33	4.05	10.50	p-Si	1.12	4.05	11.90
---	---	---	---	CuS	1.55	4.10	6.50
---	---	---	---	WSe_2_	1.62	3.80	13.80

## 3. Simulation results and discussion

### 3.1 Properties of the CIGAS absorber material

**Fig. 2:**
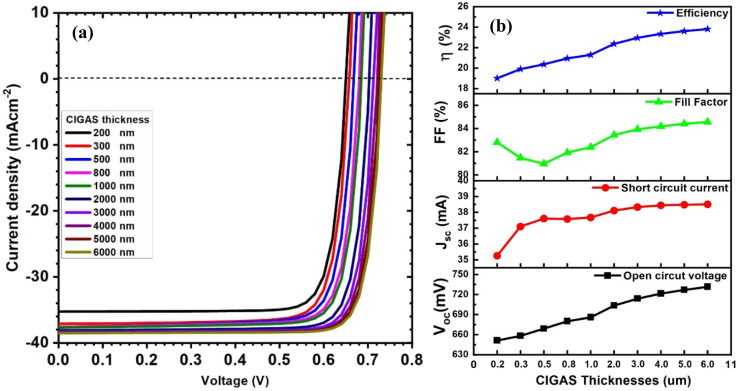
Schematic of (a) current–voltage curves and (b) curves of PV parameters for CIGAS solar cells at different CIGAS thicknesses.

Initially, the thickness of the CIGAS absorber layer was varied from 200 to 6000 nm, while keeping other layer parameters constant. Fig. 2 demonstrates the curves of current–voltage and solar cell parameters for CIGAS solar cells for CIGAS thicknesses. At a higher thickness of CIGAS absorber material, more incident photons can interact with the absorber material (according to the Beer–Lambert Law), which enhances the absorption of photons.^30^ This ensures more complete absorption across the high-energy photons from the solar spectrum due to the greater depth of absorber materials.

The increase in photon absorption with increasing CIGAS thickness can produce more photogenerated charge carriers and, as a result, increase the *J*_sc_ value. With increased production of photogenerated charge carriers, a greater probability of charge carrier collection can occur, which can improve the *V*_oc_ of solar devices. Because all solar cell parameters (*i.e.*, *J*_sc_, *V*_oc_, FF, and PCE) are interconnected, the elevated *J*_sc_ and *V*_oc_ values lead to a higher FF and efficiency of the PV device. Several publications have also reported improvements in solar cell parameters with increasing absorber material thickness.^58–60^

The results indicate that by varying the CIGAS thickness from 200 to 6000 nm, the *J*_sc_ and *V*_oc_ values changed from 35 to 38.5 mA cm^−2^ and 651 to 731 mV, respectively. Hence, the PCE of CIGAS PV devices increased from 19.01 to 23.82%. However, the PCE values obtained for CIGAS solar cells with CIGAS thickness greater than 4000 nm showed very slight variation, indicating an insignificant increase in solar cell parameters at very high thicknesses. In contrast, reduced efficiency was observed for CIGAS PV devices at a very low CIGAS thickness due to the back-contact recombination.^61^ Hence, the optimization of CIGAS thickness is a critical trade-off between performance and production cost of devices.

As mentioned earlier, the bandgap of CIGAS is tunable from 1.04 eV (for CuInSe_2_) to 2.7 eV (for CuAlSe_2_) by adjusting the gallium and aluminum content in the CIGAS material. Therefore, understanding its effect on the PV parameters is vital for optimizing the process. Because increased efficiency of PV devices is noted for the absorber material whose bandgap values are lower than 1.5 eV, the CIGAS bandgap was varied from 1.0 to 1.5 eV; however, the bandgap values for CIGAS material can be confirmed up to 1.74 eV for 10 at% of Al.^20^

**Fig. 3:**
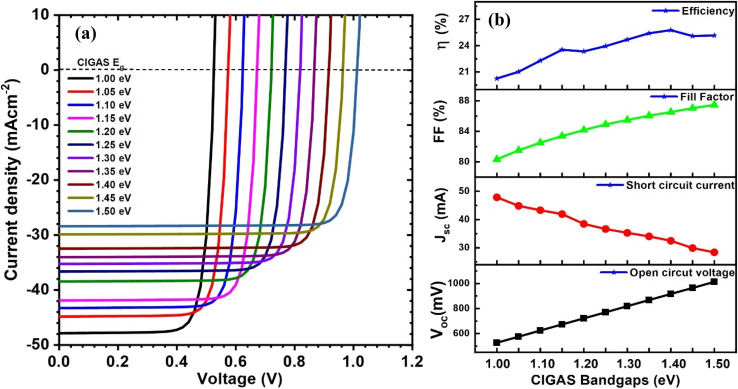
Schematic of (a) current–voltage curves and (b) curves of PV parameters for CIGAS solar cells at different CIGAS bandgaps.

Fig. 3 displays the current–voltage curves and solar cell parameters for CIGAS solar cells at different CIGAS bandgaps. With an increase in CIGAS bandgap values, all parameters were significantly varied. The *V*_oc_ value increased from 526.8 to 1013.1 mV when the CIGAS bandgap changed from 1.0 to 1.5 eV. The increase in *V*_oc_ at higher bandgap values is related to the result of higher potential differences across the solar cell, which reduces the recombination of generated electron–hole pairs.^62^ A larger bandgap requires high-energy photons to generate electron–hole pairs or excite electrons from the valence band (low-excited state) to the conduction band (high-excited state).^63^ Therefore, few photons are absorbed in higher bandgap absorber materials because the energy of photons should be equal to or greater than the bandgap of the semiconducting material. As a result, the *J*_sc_ value decreased from 47.85 (for a bandgap of 1.0 eV) to 28.40 mA cm^−2^ (for a bandgap of 1.50 eV).

It was also observed that the FF of CIGAS PV devices was enhanced at higher bandgap values, which is associated with the increase in *V*_oc_ values, even though *J*_sc_ is reduced to lower values. The PCE of devices is correlated to FF, *J*_sc_, and *V*_oc_ values, which maintain higher efficiencies with an increase in the absorber's bandgap. However, at a higher bandgap greater than 1.4 eV, the PCE began to decrease from 25.77 to 25.10%. This phenomenon is due to very low *J*_sc_ values at a higher bandgap, which reduces the efficiency of CIGAS PV devices. Various research works demonstrated similar outcomes when changing the PV parameters of solar cells at different bandgaps of absorber materials.^62–65^

**Fig. 4:**
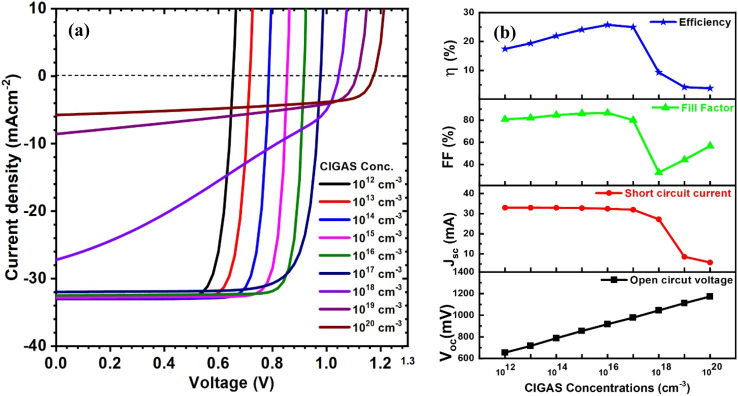
Schematic of (a) current–voltage curves and (b) curves of PV parameters for CIGAS solar cells at CIGAS carrier concentrations ranging from 10^12^ to 10^20^ cm^−3^.

The simulation study then focused on optimizing the carrier concentration of the CIGAS absorber layer. Initially, the CIGAS carrier concentration was examined between 10^12^ and 10^20^ cm^−3^ (see Fig. 4), and the other parameters were fixed. All PV parameters drastically changed at different CIGAS carrier concentrations. This variation in PV parameters is based on the deviation in electric field intensity, as well as the space charge region of CIGAS solar cells.^64^ When the CIGAS concentrations increased from 10^12^ to 10^20^ cm^−3^, the open-circuit voltage was enhanced from 655 to 1174 mV, and the short circuit current density was diminished from 33 to 6 mA cm^−2^. Higher CIGAS carrier concentrations drive a larger *V*_oc_ value by decreasing the emitter saturation current density and providing a greater built-in electric field. Conversely, the semiconducting material at a higher carrier concentration can shift into a metallic conductive state, which decreases the space charge region of the PV device.^30^

This phenomenon supports band-to-band recombination, interface recombination, and coulombic interactions of generated charge carriers. As a result, the efficiency of charge carrier extraction and the *J*_sc_ value degrade at greater CIGAS carrier concentrations. Finally, the FF and the efficiency of CIGAS PV devices are greatly influenced. The PCE initially increased from 17.4 to 25.8% when the carrier concentration of CIGAS was augmented from 10^12^ to 10^16^ cm^−3^. Beyond 10^16^ cm^−3^, the efficiency sharply declined to 3.8%, driven by the collapse of the *J*_sc_ value.

**Fig. 5:**
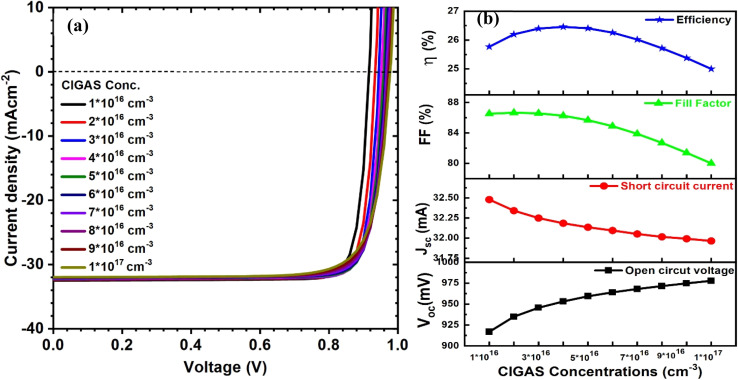
Schematic of (a) current–voltage curves and (b) curves of PV parameters for CIGAS solar cells at CIGAS carrier concentrations ranging from 10^16^ to 10^17^ cm^−3^.

This theoretical modeling was then focused on CIGAS carrier concentration from 10^16^ to 10^17^ mA cm^−2^ to determine the best conditions for achieving the highest efficiency for CIGAS PV devices. Fig. 5 shows the curves of current–voltage and solar cell parameters of CIGAS thin film solar cells at CIGAS carrier concentrations ranging from 10^16^ to 10^17^ cm^−3^. Similar results were obtained with an increase in CIGAS carrier concentration from 10^16^ to 10^17^ cm^−3^. The highest PCE of 26.5% was observed at 4 × 10^16^ cm^−3^; thereafter, the efficiency begins to decrease to a lower value through the reduction of the *J*_sc_ value. Similar effects of the absorber's carrier concentration on PV parameters have been widely reported in the literature.^66–68^

### 3.2 Properties of CdS buffer material

**Fig. 6:**
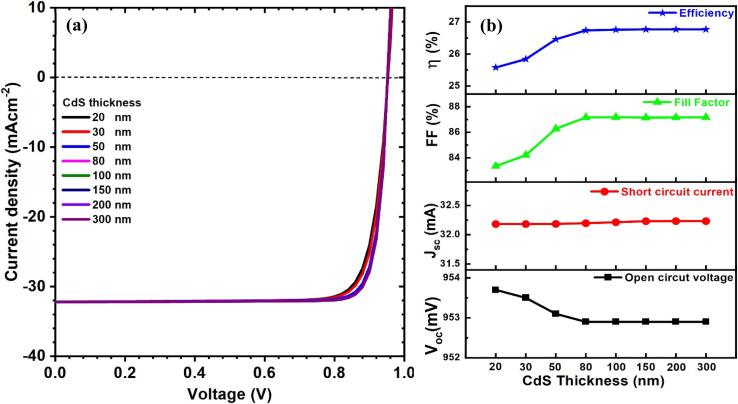
Schematic of (a) current–voltage curves and (b) curves of PV parameters for CIGAS solar cells at different CdS thicknesses.

The properties of CdS were simulated, as it is considered a vital material in p–n junction formation for CIGAS PV devices. CdS buffer material has a direct bandgap of 2.45 eV that allows the incident photons to pass through to the absorber layer. First, the CdS thickness was varied from 20 to 300 nm. Fig. 6 displays the curves of current–voltage and solar cell parameters for CIGAS thin film solar cells at different CdS thicknesses. The simulation results showed that there is only a minor impact of CdS thickness on the solar cell parameters. Because the CdS thickness is increased from 20 to 300 nm, the open-circuit voltage drops from 953.7 to 952.9 mV, while the short circuit current density remained constant.

Despite the slight drop in *V*_oc_, these results suggest a notable increase in the FF from 83 to 87% and PCE from 25.6 to 26.8%, which is attributed to the improvement in the quality of CdS material at a higher thickness. However, a very high thickness of the CdS buffer layer in the CIGSe solar cell negatively affected the PCE by absorbing photons that would otherwise be absorbed by the CIGAS layer, which increases the series resistance of the device.^69^ The reduced efficiency at lower CdS thicknesses (less than 80 nm) was attributed to a decrease in the shunt resistance value, which directly affects the FF and, consequently, the PCE of the CIGAS PV devices.^70,71^ Therefore, the CdS buffer layer must be sufficiently thin without defects to obtain high device performance. Herein, a CdS thickness of 80 nm was considered an optimized condition, where performance gains began to saturate when the thickness was greater than 80 nm.

**Fig. 7:**
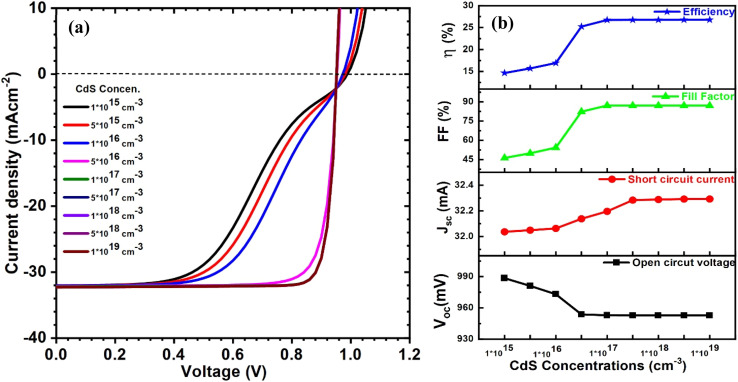
Schematic of (a) current–voltage curves and (b) curves of PV parameters for CIGAS solar cells at different CdS concentrations.

After that, the CdS concentration was changed from 10^15^ to 10^19^ cm^−3^, while other parameters remained constant. The curves of current–voltage and solar cell parameters for CIGAS thin film solar cells are presented in Fig. 7. From the simulation results, all PV parameters except short circuit current density were profoundly influenced after varying the carrier concentration of CdS. A slight improvement in efficiency from 15 to 17% was observed when the CdS carrier concentration increased from 10^15^ to 10^16^ cm^−3^. For CdS carrier concentrations greater than 10^16^ cm^−3^, the efficiency of CIGAS PV devices significantly increased from 17 to 26.8%.

Progressive efficiency at carrier concentrations greater than 10^16^ cm^−3^ is related to a higher CdS carrier concentration as compared to the CIGAS carrier concentration (*i.e.*, 4 × 10^16^ cm^−3^). The collection of created charge carriers is advanced at higher CdS concentrations by increasing the space-charge region toward the CIGAS absorber layer.^30^ Therefore, increasing the CdS carrier concentration reduces the barrier potential at the CdS/CIGAS junction, as a result of lowering the interface recombination.^72^ However, the CdS carrier concentration is less than the CIGAS carrier concentration and has the ability to absorb incident photons at the CdS layer. This decreases the amount of incident photons absorbed in the absorber layer and lowers the PCE. The identical and highest PCE of 26.8% was observed at the CdS carrier concentration of 5 × 10^17^ cm^−3^ and above. Various studies reported similar outcomes related to solar cell parameters after varying CdS buffer layer carrier concentrations.^72–74^

### 3.3 Properties of ZnO window material

Similar to CdS buffer material, ZnO material is considered very suitable for achieving high performance of CIGAS PV devices due to its characteristics, such as high n-type conductivity and mobility, excellent thermal and mechanical stability, wide bandgap, and low cost. ZnO also allows light to pass towards the absorber layer, thereby enabling efficient generation and collection of charge carriers.

**Fig. 8:**
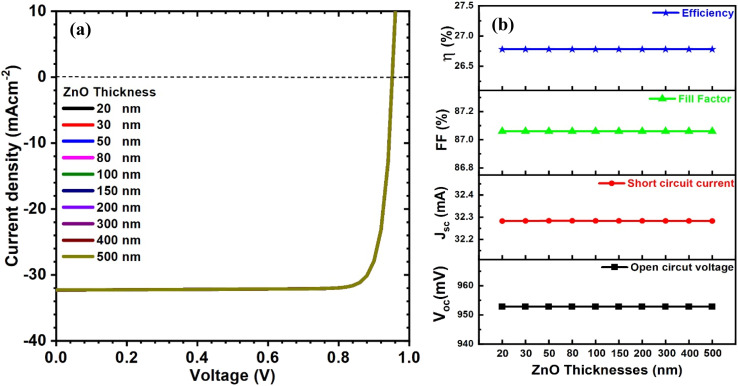
Schematic of (a) current–voltage curves and (b) curves of PV parameters for CIGAS solar cells at different ZnO thicknesses.

**Fig. 9:**
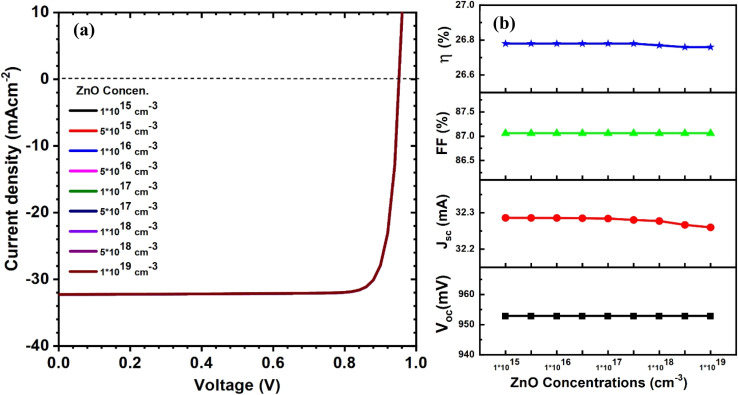
Schematic of (a) current–voltage curves and (b) curves of PV parameters for CIGAS solar cells at different ZnO concentrations.

As a part of the optimization process, we systematically varied ZnO layer thickness from 20 to 500 nm and the carrier concentration from 10^15^ to 10^19^ cm^−3^ to investigate their effects on PV parameters. The curves of current–voltage and solar cell parameters for CIGAS PV devices at different thicknesses and carrier concentrations of ZnO material are displayed in Fig. 8 and 9, respectively.

By analyzing the simulation results, all the PV parameters remained unchanged for both cases. It was noted that the properties of ZnO do not significantly affect the PV parameters because ZnO is situated far from the p–n junction. Several studies have reported the effects of the properties of ZnO on the PCE of CIGAS PV devices.^75–77^ However, a thick ZnO layer or a ZnO carrier concentration lower than that of the absorber layer can reduce the device performance by enhancing the series resistance, which directly influences the FF and the absorption of light in it. Therefore, thin high-quality ZnO material and high ZnO carrier concentration are preferred to achieve greater efficiency for CIGAS thin film solar cells.

### 3.4 Properties of BSF material

The BSF material is an extra layer placed between the CIGAS absorber layer and back contact. Most of the metals used as a back contact in CIGAS PV devices form Schottky-barrier contact that can impede the transportation of charge carriers. The use of the BSF layer is intended to create an ohmic contact with the CIGAS absorber layer, thereby minimizing the back-contact recombination.^78^ The BSF layer can enhance charge carrier collection, which significantly increases the PCE of CIGAS PV devices compared to conventional solar cells.

**Fig. 10:**
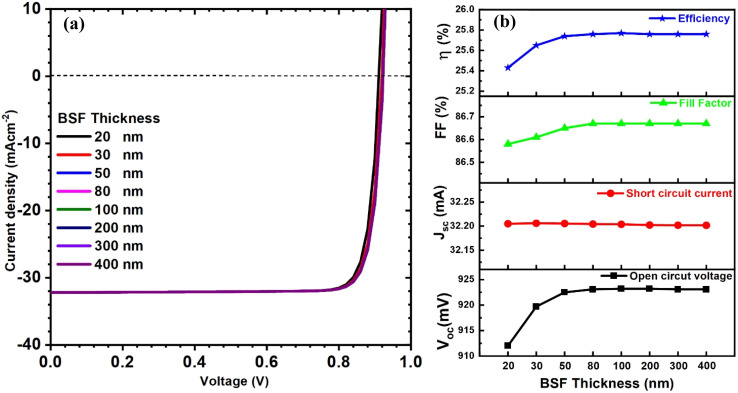
Schematic of (a) current–voltage curves and (b) curves of PV parameters for CIGAS solar cells at different BSF thicknesses.

The suitable properties of the BSF layer must be optimized to realize this layer's benefit to the solar cell structure. Initially, the influence of BSF thickness on the PV parameters was investigated. Fig. 10 shows the current–voltage curve and solar cell parameters for CIGAS thin film solar cells at different BSF layer thicknesses. It was observed that increasing the BSF thickness from 20 to 400 nm led to an increase in open-circuit voltage from 912 to 923 mV at a carrier concentration of 10^16^ cm^−3^. The short circuit current density remained unchanged with an increase in BSF thickness.

The thicker BSF material promotes the absorption of more photons in the devices, which allows the generation and collection of additional charge carriers. Conversely, a thinner BSF material reduces the collection of charge carriers and advances the rear recombination by decreasing the shunt resistance.^79,80^ As a result, the PCE of CIGAS PV devices is improved with an increase in BSF thickness. These results are in accordance with several published research works.^51–56^ The efficiency value remained constant for BSF thicknesses greater than 80 nm, and hence, it is considered as an optimized BSF thickness with an efficiency of 25.8% for CIGAS thin film solar cells.

**Fig. 11:**
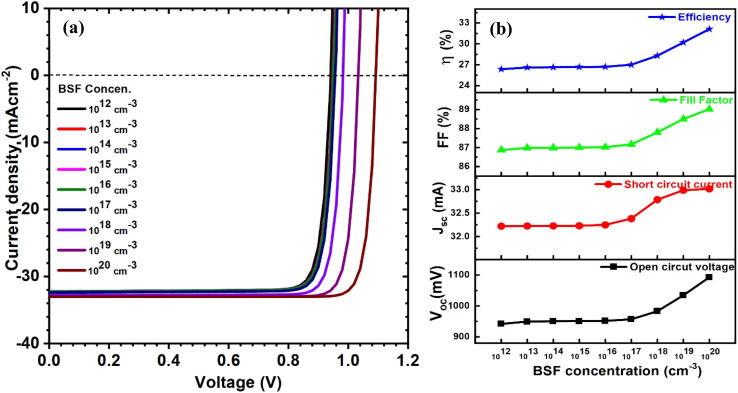
Schematic of (a) current–voltage curves and (b) curves of PV parameters for CIGAS solar cells at different BSF concentrations.

We then concentrated on the carrier concentration of the BSF layer, which varied from 10^12^ to 10^20^ cm^−3^. The curves of current–voltage and solar cell PV parameters for CIGAS solar cells at different BSF carrier concentrations are presented in Fig. 11. It was noted that all PV parameters except short circuit current density were significantly improved. Increasing the BSF carrier concentration from 10^12^ to 10^20^ cm^−3^ enhanced the open-circuit voltage from 942 to 1093 mV and the PCE from 26 to 32% in CIGAS solar cells.

This substantial increase in PV parameters, particularly *V*_oc_ and PCE, at higher carrier concentrations of the BSF layer is due to the strong electric field induced in the layer, which repels the minority charge carriers (*i.e.*, electrons in p-type semiconductors) away from the back-contact surface. Thus, the higher BSF carrier concentrations facilitate the collection of charge carriers by reducing the recombination losses and series resistance of solar cells.^81^ Conversely, lower BSF carrier concentrations provide weaker field effects that can decrease the band bending and built-in potential of PV devices.^82^ Consequently, the open-circuit voltage is reduced due to higher back-contact recombination. Herein, the highest efficiency of 32% was observed for a BSF carrier concentration of 10^20^ cm^−3^ and is considered as an optimized condition. Therefore, this study complies with the previously published literature.^51–56^

### 3.5 Optimized results

**Fig. 12:**
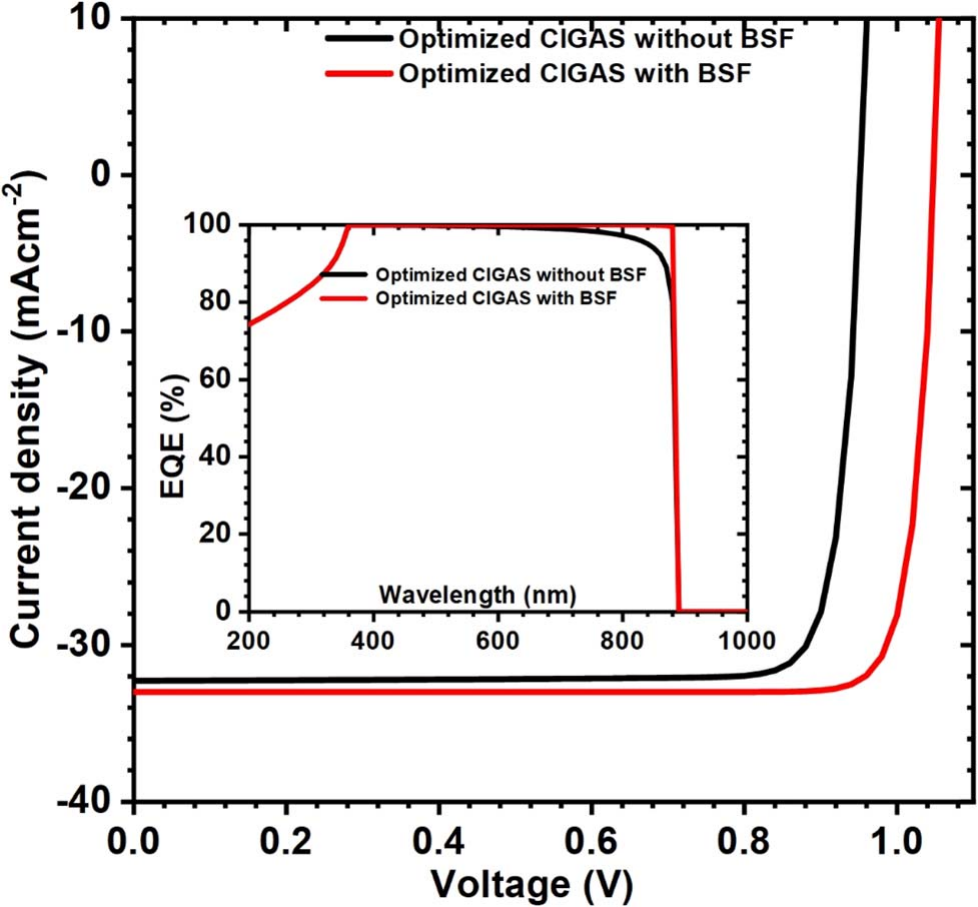
Schematic of current–voltage curves inserted with quantum-efficiency curves and PV parameters for optimized CIGAS solar cells with and without BSF materials.

In this section, the optimized properties of CIGAS absorber material, CdS buffer material, ZnO window layer, and BSF material were employed to obtain the maximum efficiency under these idealized conditions. Fig. 12 shows the current–voltage curves for optimized CIGAS PV devices, with an inset displaying quantum-efficiency curves with and without BSF materials. For the CIGAS absorber material, the most optimal results were found at a thickness of 4000 nm, bandgap of 1.4 eV, and carrier concentration of 4 × 10^16^ cm^−3^.

A thickness of 80 nm and carrier concentration of 5 × 10^17^ cm^−3^ for CdS provide maximum efficiency for the CIGAS thin film solar cells. The properties of ZnO did not show significant deviation in PV parameters; however, the properties of ZnO that enable the achievement of high-efficiency devices are compatible with the properties of CdS. Moreover, the BSF material's optimized thickness and carrier concentration were 50 nm and 10^20^ cm^−3^, respectively. After using the BSF layer, the PV parameters, such as *V*_oc_, *J*_sc,_ and FF, were enhanced from 953 to 1049 mV, 32 to 33 mA cm^−2^, and 87 to 88.7%, respectively.

Finally, optimum efficiencies of 26.8 and 30.7% were observed for CIGAS PV devices with and without BSF material, respectively. Higher efficiency for CIGAS PV devices with a BSF layer was correlated with a decrease in back surface recombination by the formation of an ohmic contact to transport the generated charge carriers. This statement is also supported by the QE curve with almost a square nature (ideal quantum efficiency), indicating that the BSF layer in CIGAS PV devices can improve the absorption of long-wavelength photons and diffusion length.

### 3.6 Different buffer materials

After the optimization of the CIGAS PV devices, the theoretical modeling was extended to analyze the effects of different types of buffer materials whose properties are completely different. The materials, such as CdS, In_2_S_3_, ZnS, ZnSe, In_2_Se_3_, V_2_O_5_, ZnO, and MgZnO, are introduced as buffer window layers in CIGAS PV devices.^48–50^ The thickness and carrier concentration values are the same as previously optimized properties of buffer materials. The other properties, namely bandgap, electron affinity, and dielectric permittivity, were assigned according to the respective values of each buffer material.

**Fig. 13:**
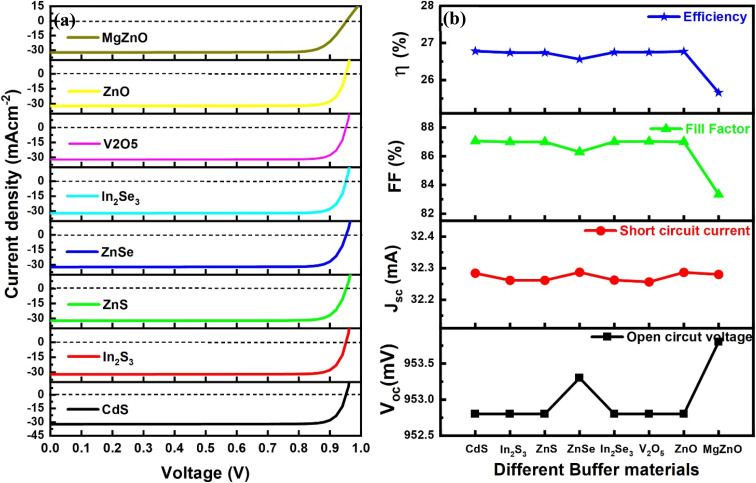
Schematic of (a) current–voltage curves and (b) curves of PV parameters for CIGAS solar cells for different buffer materials.

Fig. 13 displays the curves of current–voltage and PV parameters for CIGAS thin film solar cells for different buffer window materials. These results showed that PCE values greater than 25% were observed for CIGAS PV devices using all buffer materials. The highest *V*_oc_ of 953.8 mV was observed for the MgZnO buffer material, and the highest efficiency of 26.78% was observed for the CdS buffer material. Therefore, all these buffer materials except MgZnO can offer more than 26% efficiency and are suitable to use as buffer layers in PV devices. However, properties of buffer materials, such as lattice mismatch, conductivity, mechanical and chemical stability, availability, stability against photocorrosion, and cost-effectiveness, must be considered to increase our understanding of their effects on solar cell parameters.^83,84^

### 3.7 Different BSF materials

**Fig. 14:**
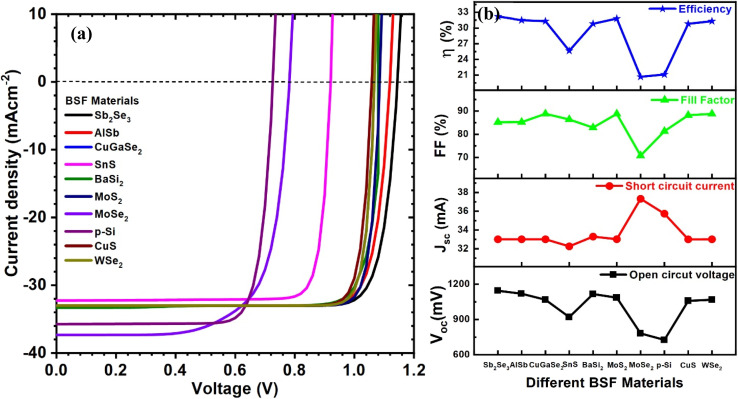
Schematic of (a) current–voltage curves and (b) curves of PV parameters for CIGAS solar cells for different BSF materials.

The influence of different types of BSF materials on the PV parameters was also simulated. Various materials, such as Sb_2_Se_3_, AlSb, CuGaSe_2_, SnS, BaSi_2_, MoS_2_, MoSe_2_, p-Si, CuS, and WSe_2_, are promising as back surface field layers for CIGAS thin film solar cells.^51–56^ The graphs of current–voltage and PV parameters for CIGAS solar cells for different BSF materials are displayed in Fig. 14. Similarly, the optimized thickness and carrier concentration from the above results of the BSF layer were used for all BSF materials.

From the results, the lowest and highest *V*_oc_ of 727 and 1145 mV were observed for p-Si and Sb_2_Se_3_ BSF materials, respectively. The *J*_sc_ value varied from 32.3 (for SnS) to 37.3 mA cm^−2^ (for MoSe_2_). With the exception of BSF materials such as SnS, MoSe_2,_ and p-Si, all tested BSF materials achieved greater than 30% efficiency for CIGAS PV devices, with the highest efficiency of 32.2% for the Sb_2_Se_3_ BSF layer.

These results demonstrated that many of the above-mentioned BSF materials are favorable for CIGAS solar cells. However, various properties of these materials can be studied for further optimization processes. The application of a BSF layer enhanced the PCE of CIGAS PV devices by up to 32%. This outcome also verified the advantage of utilizing BSF to increase efficiency by more than 5% compared to normal solar cells. Therefore, after comparing the solar cell PV parameters to other BSF materials, Sb_2_Se_3_ was considered the best BSF material for CIGAS thin film solar cells.

### 3.8 Analysis of experimental parameters

All the above results were obtained by analyzing the theoretical parameters of each layer used in CIGAS PV devices. Herein, the experimentally obtained parameters of materials were introduced in the SCAPS software. Because the PV parameters are strongly affected by the properties of the absorber and buffer layers, only the experimental parameters of these layers were considered. Table 3 presents the experimentally obtained parameters for CIGAS materials (*i.e.*, thickness and bandgap) and for CdS materials (*i.e.*, thickness, bandgap, mobility, and carrier concentration). These parameters were replaced in theoretically optimized conditions of CIGAS PV devices, which were obtained from previously published literature.^85,86^ The CIGAS and CdS were synthesized from pulsed laser deposition and chemical bath deposition, respectively.

**Table 3:** Experimental parameters obtained for CIGAS and CdS materials^20,85,86^

Parameters samples	Thickness (nm)	Bandgap (eV)	Mobility (cm^2^ V^−1^ s^−1^)	Carrier concentration (cm^−3^)
CIGAS 1	∼200	1.22	—	—
CIGAS 2	∼200	1.35	—	—
CIGAS 3	∼200	1.37	—	—
CIGAS 4	∼200	1.45	—	—
CIGAS 5	∼200	1.47	—	—
CdS 1	∼40	2.49	116.00	4.5 × 10^14^
CdS 2	∼80	2.54	15.00	1.9 × 10^16^
CdS 3	∼130	2.55	31.00	7.2 × 10^15^
CdS 4	∼160	2.58	16.00	5.6 × 10^14^
CdS 5	∼170	2.64	74.00	1.0 × 10^16^

#### 3.8.1 Experimental parameters of CIGAS

**Fig. 15:**
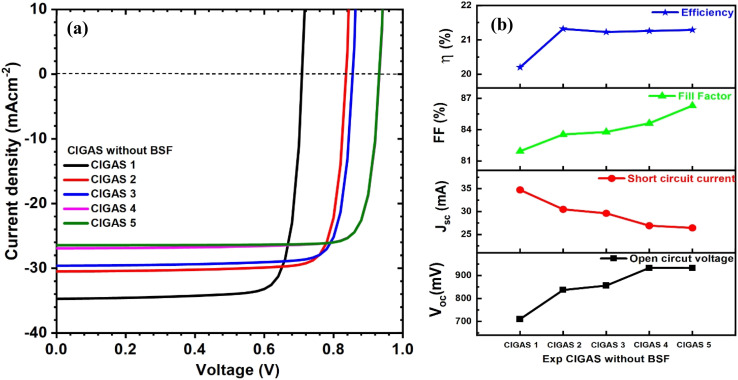
Schematic of (a) current–voltage curves and (b) curves of PV parameters for CIGAS solar cells, fitting the experimental parameters for CIGAS without BSF materials.

**Fig. 16:**
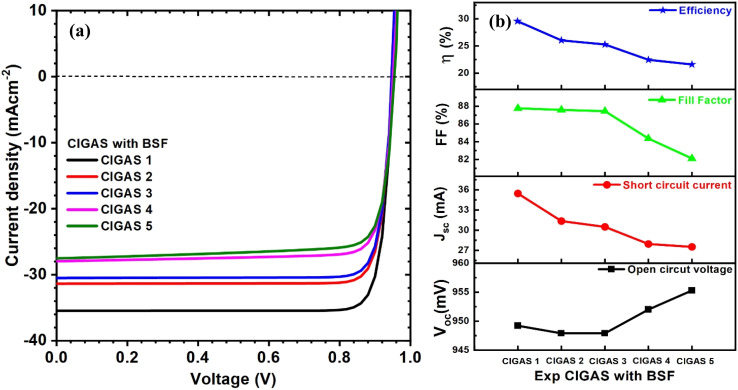
Schematic of (a) current–voltage curves and (b) curves of PV parameters for CIGAS solar cells, fitting experimental parameters for CIGAS with BSF materials.

Herein, the experimental parameters of the CIGAS absorber material, such as thickness and bandgap, were introduced. The bandgap of CIGAS was varied from 1.22 to 1.47 eV, but the thickness of the CIGAS thin film remained constant. Thus, the CIGAS PV devices with and without a BSF layer were specifically analyzed at different bandgaps of CIGAS, as shown in Fig. 15 and 16, respectively. The simulation results confirmed that all the PV parameters were influenced.

As mentioned earlier, the bandgap of the CIGAS absorber layer enhances the *V*_oc_ by forming higher potential differences across the solar cells.^62^ The *J*_sc_ value is reduced by decreasing the generated charge carriers at higher band gaps.^63^ However, the bandgap of CIGAS showed two different results for the FF and PCE. The FF increased from 82 to 86% and decreased from 87.8 to 82% for CIGAS thin film solar cells without and with a BSF layer, respectively. There was no significant variation in the efficiency of CIGAS PV devices without a BSF layer (*i.e.*, approximately 21%). Conversely, the efficiency was reduced from 29.5 to 21.6% for CIGAS thin film solar cells with a BSF layer.

The efficiencies of CIGAS PV devices with BSF are comparatively higher than PV devices without a BSF material.^51^ This result confirms that absorber material properties influence the PV parameters, while the BSF material also plays a significant role in altering the outcomes compared to standard conditions. Maximum efficiency will be achieved with CIGAS material with a thickness of 200 nm and bandgap of 1.22 eV (*i.e.*, CIGAS 1).

#### 3.8.2 Experimental parameters of CIGAS and CdS

**Fig. 17:**
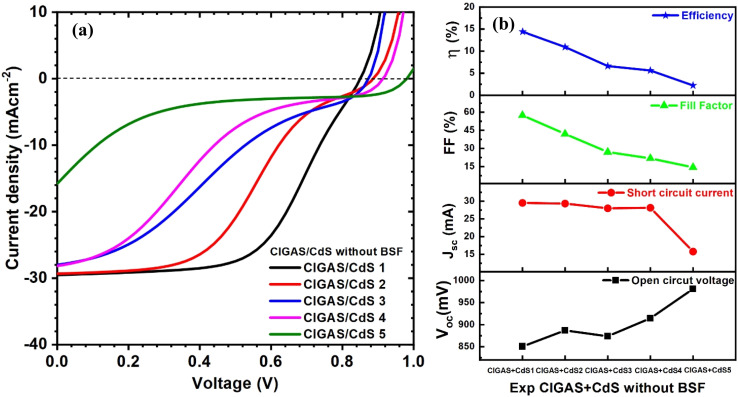
Schematic of (a) current–voltage curves and (b) curves of PV parameters for CIGAS solar cells, fitting experimental parameters for CIGAS and CdS without BSF materials.

**Fig. 18:**
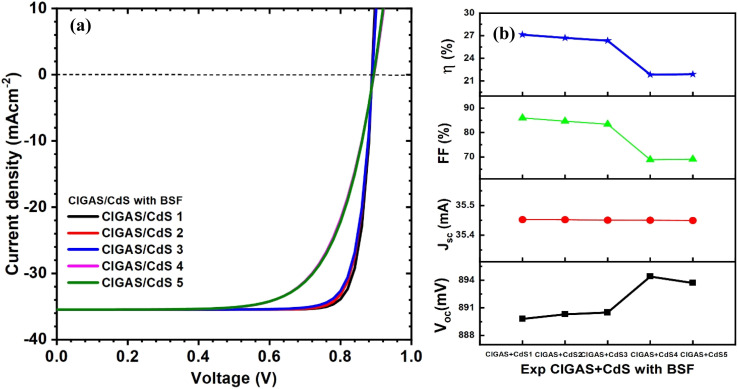
Schematic of (a) current–voltage curves and (b) curves of PV parameters for CIGAS solar cells, fitting experimental parameters for CIGAS and CdS with BSF materials.

Finally, the experimentally obtained properties of CIGAS and CdS were simulated to understand their effect on the solar cell parameters. The CdS thin films were synthesized using the chemical bath deposition technique with different deposition times and a constant temperature of 80 °C. Therefore, the thickness of CdS increased from 40 to 170 nm. The curves of current–voltage and PV parameters for CIGAS PV devices without and with a BSF layer are correspondingly displayed in Fig. 17 and 18. For both cases, the efficiencies of CIGAS PV devices are relatively lower when experimental parameters are applied to both CIGAS and CdS materials, than when applied only to CIGAS.

It was observed that the properties of CdS directly impacted the FF and PCE of CIGAS thin film solar cells. When the deposition time of CdS thin films increased from 20 min to 60 min, the efficiency of PV devices without and with the BSF layer was independently reduced from 14.4 to 2.2% and from 27.1 to 21.9%, respectively. The use of a BSF layer in CIGAS thin film solar cells achieved higher efficiency as compared to the structure without one.^51^ This result confirmed that the BSF layer is a vital material to improve the PCE for CIGAS PV devices.

Although there is variation in the properties of CdS thin films with deposition time, the decrease in efficiency at a higher deposition time is related to the thickness of the CdS buffer layer. A higher thickness of CdS can reduce the passing of incident photons toward the CIGAS absorber layer and increase the series resistance that directly influences the fill factor of PV devices.^87^ Therefore, the first two conditions (*i.e.*, CIGAS + CdS 1 and CIGAS + CdS 2) are suitable to employ in CIGAS thin film solar cells based on their performance.

## 4. Conclusions

CIGAS PV devices with novel materials and structures were effectively investigated through the SCAPS-1D software in this work. Initially, the numerical analysis was carried out for the optimization process for the CIGAS PV device by analyzing the properties (*i.e.*, thickness and carrier concentration) of the CIGAS absorber, CdS buffer, ZnO window, and BSF materials. The thickness of materials drastically influenced the PV parameters by varying the absorption of photons as well as the series resistance of solar cells. However, the carrier concentration of each material can assist in understanding the carrier lifetime and diffusion length of the carriers through recombination mechanisms. These parameters were adjusted for each material in such a way that the efficiency of CIGAS devices was as high as possible.

The simulation results showed that the optimized efficiency was comparatively higher for CIGAS devices with a BSF layer (*i.e.*, 30.7%) than without a BSF layer (*i.e.*, 26.8%). This increase in the performance of CIGAS devices with a BSF layer was related to the decrease in the back-contact recombination by favoring ohmic contact with the CIGAS absorber layer. After analyzing the various types of buffers and BSF materials, CdS and Sb_2_Se_3_ demonstrated their suitability as materials for a buffer layer and a BSF layer for obtaining efficient CIGAS PV devices, respectively. At last, the properties of the experimentally obtained CIGAS absorber and CdS buffer materials were inserted into theoretically optimized conditions to understand the CIGAS device performance. It evidently confirmed that the thickness of CdS and the use of the BSF layer can significantly enhance the PCE of CIGAS PV devices.

In this simulation study, we designed CIGS-based solar cells with new CIGAS absorber materials, and also studied the properties of each material used in CIGAS solar cells. Hence, this investigation can assist in the design of novel solar cell configurations by reducing cost and time, thus providing a base for experimental activities.

## Conflicts of interest

The authors declare that they have no conflict of interest.

## Data Availability

The authors confirm that the data supporting the findings of this study are available within the article. The raw data that support the findings of this study are available from the corresponding authors upon reasonable request.
